# Discontinuities in quinoa biodiversity in the dry Andes: An 18-century perspective based on allelic genotyping

**DOI:** 10.1371/journal.pone.0207519

**Published:** 2018-12-05

**Authors:** Thierry Winkel, María Gabriela Aguirre, Carla Marcela Arizio, Carlos Alberto Aschero, María del Pilar Babot, Laure Benoit, Concetta Burgarella, Sabrina Costa-Tártara, Marie-Pierre Dubois, Laurène Gay, Salomón Hocsman, Margaux Jullien, Sara María Luisa López-Campeny, María Marcela Manifesto, Miguel Navascués, Nurit Oliszewski, Elizabeth Pintar, Saliha Zenboudji, Héctor Daniel Bertero, Richard Joffre

**Affiliations:** 1 Centre d'Écologie Fonctionnelle et Évolutive CEFE, Institut de Recherche pour le Développement IRD, CNRS, Université de Montpellier, Université Paul-Valéry Montpellier UPVM3, École Pratique des Hautes Études EPHE, Montpellier, France; 2 Facultad de Ciencias Naturales e Instituto Miguel Lillo, Universidad Nacional de Tucumán (FCN e IML, UNT), San Miguel de Tucumán, Argentina; 3 Instituto de Recursos Biológicos CIRN-INTA, Hurlingham, Buenos Aires, Argentina; 4 Instituto Superior de Estudios Sociales, Consejo Nacional de Investigaciones Científicas y Técnicas (ISES, CONICET), San Miguel de Tucumán, Argentina; 5 Instituto de Arqueología y Museo, Facultad de Ciencias Naturales e Instituto Miguel Lillo, Universidad Nacional de Tucumán (IAM, FCN e IML, UNT), San Miguel de Tucumán, Argentina; 6 Centre d'Écologie Fonctionnelle et Évolutive CEFE, CNRS, Université de Montpellier, UPVM3, EPHE, IRD, Montpellier, France; 7 UMR AGAP Amélioration Génétique et Adaptation des Plantes Méditerranéennes et Tropicales, CIRAD, INRA, SupAgro, Montpellier, France; 8 Departamento de Tecnología, Universidad Nacional de Luján, Luján, Buenos Aires, Argentina; 9 UMR AGAP Amélioration Génétique et Adaptation des Plantes Méditerranéennes et Tropicales, INRA, CIRAD, SupAgro, Montpellier, France; 10 Centre de Biologie pour la Gestion des Populations CBGP, INRA, IRD, CIRAD, SupAgro, Montpellier, France; 11 Institut de Biologie Computationnelle IBC, Montpellier, France; 12 Social Sciences Division, Austin Community College, Austin, Texas, United States of Amrica; 13 Cátedra de Producción Vegetal, Facultad de Agronomía, Universidad de Buenos Aires, and IFEVA-CONICET, Buenos Aires, Argentina; Brigham Young University, UNITED STATES

## Abstract

History and environment shape crop biodiversity, particularly in areas with vulnerable human communities and ecosystems. Tracing crop biodiversity over time helps understand how rural societies cope with anthropogenic or climatic changes. Exceptionally well preserved ancient DNA of quinoa (*Chenopodium quinoa* Willd.) from the cold and arid Andes of Argentina has allowed us to track changes and continuities in quinoa diversity over 18 centuries, by coupling genotyping of 157 ancient and modern seeds by 24 SSR markers with cluster and coalescence analyses. Cluster analyses revealed clear population patterns separating modern and ancient quinoas. Coalescence-based analyses revealed that genetic drift within a single population cannot explain genetic differentiation among ancient and modern quinoas. The hypothesis of a genetic bottleneck related to the Spanish Conquest also does not seem to apply at a local scale. Instead, the most likely scenario is the replacement of preexisting quinoa gene pools with new ones of lower genetic diversity. This process occurred at least twice in the last 18 centuries: first, between the 6th and 12th centuries—a time of agricultural intensification well before the Inka and Spanish conquests—and then between the 13th century and today—a period marked by farming marginalization in the late 19th century likely due to a severe multidecadal drought. While these processes of local gene pool replacement do not imply losses of genetic diversity at the metapopulation scale, they support the view that gene pool replacement linked to social and environmental changes can result from opposite agricultural trajectories.

## Introduction

The Andes, a global hotspot of past and present crop biodiversity, has witnessed huge environmental and socio-cultural changes, including the climatic fluctuations of the late Holocene and the disruption of native societies following the Spanish Conquest [1–3]. Less dramatically, progressive changes in agricultural knowledge and practices have ensured the resilience of Andean societies to date [4–6]. Amid these historical changes, several Andean-origin crops have diversified and were successfully disseminated throughout the world, such as tomato (S*olanum lycopersicum*), potato (*S*. *tuberosum*), beans (*Phaseolus* spp.), chiles (*Capsicum* spp.) and, more recently, quinoa (*Chenopodium quinoa*) [7].

**Fig 1:**
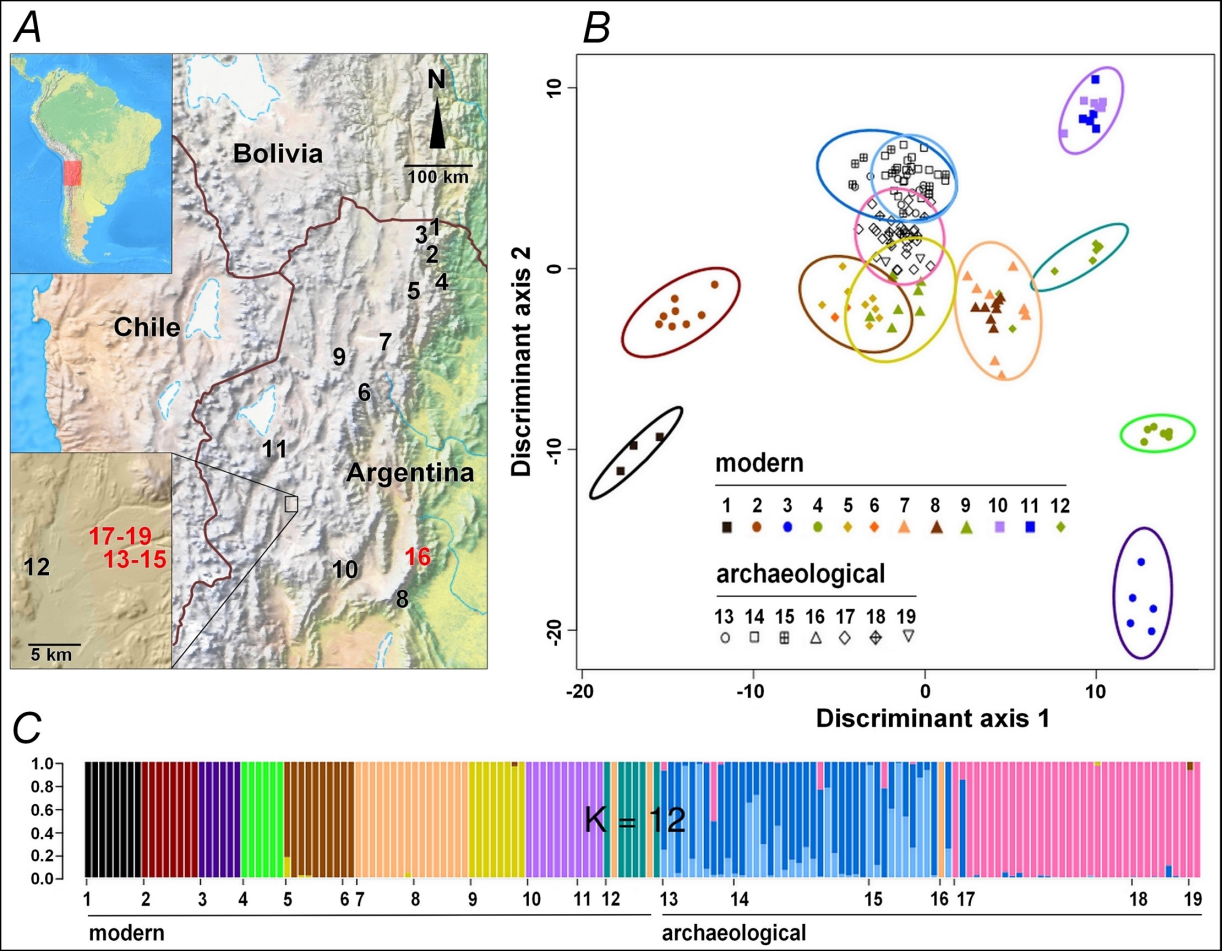
Geographic localization and genetic classification of ancient and modern quinoas collected in Northwest Argentina. (*A*): Map of the dry Andes localizing ancient and modern quinoa samples (red and black numbers, respectively; detailed sample description in Table B in S1 Appendix). (*B*): Scatterplot of the Discriminant Analysis of Principal Components (DAPC). Individuals are represented by symbols according to their sample of origin; colored inertia ellipses define the clusters identified with a k-means algorithm for k = 12. (*C*): Individual assignment probability to each cluster from DAPC. Horizontal axis shows the sample codes as in *A* and *B*; vertical axis shows the assignment probabilities for k = 12.

In the Central Andes of Peru and northern Bolivia, the rise and fall of past agrarian societies due to political and environmental changes seems a most likely scenario [8–10]. But in the dry Andes of Northwest Argentina, southern Bolivia and northern Chile (Fig 1A), a different historical trajectory took place due to the relative importance of pastoralism versus agriculture [11] (Table A in S1 Appendix). Around 5000 BP (years before present) pastoralism arose among local hunter-gatherers who had been established in the region since 12000 BP [12,13]. These hunter-gatherers in transition to food production also developed crop planting early in the dry Andes, as evidenced by remains of plant domesticates dating back *ca* 5000 BP [14,15]. Farming was a productive practice in the region at that time and until the Inka period and the Spanish conquest, though without reaching a comparable level of significance to that observed in the Central Andes [16, 17]. Then, at a still uncertain time during the Colonia and early Republic periods (*viz*. 16th to 19th centuries), agrarian systems in the most arid highlands reverted to a primarily pastoralist economy, wherein small-scale crop farming assumed a limited role, a situation that persists today [18]. Palaeoecological studies revealed that substantial Holocene fluctuations in the regional climate likely coincided with these socio-historical changes [19–22]. Two relatively humid periods (12000–8000 BP and 5000–1500 BP) alternated with drier periods (8000–5000 BP and 1500 BP to the Present); during the dry phases water resources concentrated in some valleys and basins [23].

The question then arises as to how these social and environmental changes affected local crop biodiversity throughout this period. Specifically, have climatic and agrarian changes—and their related transformations in social structures and local economy—led to genetic changes in native crop species? The quinoa crop in the dry Andes of Argentina provides a case study for investigating these issues since local conditions of low temperature and air dryness allowed for the conservation of abundant biological material in what once were residential places, granaries or tombs [24,25]. In modern quinoa samples, molecular markers reveal a diversity essentially shaped by broad biogeographic features separating—among others—quinoas from temperate highlands, arid highlands, mid- and high-altitude valleys, and western versus eastern lowlands [26]. Molecular genotyping, applied to ancient samples, should thus allow for tracking quinoa biodiversity in space and time, providing a new tool to investigate the agrarian economy of past societies and go further in-depth into the history of human-plant relationships [27].

Analyzing genetic markers within a coalescence framework, we track changes and continuities in quinoa diversity in the dry Andes over the last two millennia. Coalescence theory allows to identify the most probable trajectory among the many possible genealogies in a regional gene pool [28]. Then we discuss how natural and human circumstances paralleling these temporal patterns in genetic diversity could explain them. Our archaeological study sites are located in cold and arid highlands, with one site in a mesothermic Andean valley located at the same latitude (Fig 1A, Table B in S1 Appendix) [12,14,17,29]. They provided well-preserved quinoa seeds, with a broad chronological range spanning the time of early husbandry (*ca* 1800 BP), to periods of stable agro-pastoralist societies (*ca* 1400 BP) and complex corporative societies (*ca* 800–700 BP). To evaluate the relationship of these ancient quinoas with the present-day germplasm, we studied a reference panel of quinoas collected in 2006–2007 from different environments in the Andean highlands of Argentina [26,30] (Fig 1A, Table B in S1 Appendix). Some archaeological sites supplied both dark and white seeds, which allowed us to explore the diversity of cultivated quinoa (generally white-seeded) and their weedy relatives (all dark-seeded).

## Results

Genetic diversity, selfing rates and genetic structure were studied from 157 quinoa seeds (76 ancient, 81 modern) genotyped at 24 microsatellite loci (see Section C in S1 Appendix for detailed results).

### Genetic diversity and selfing rate

Estimates of the diversity indices and the selfing rates of the 19 populations sampled are summarized in Table E in S1 Appendix. Expected heterozygosity (*He*) in the subset of modern quinoas showed highly variable values (range 0.02–0.70), consistent with those found in an independent study on the same samples [26]. Similarly, selfing rates (*s(F*_*is*_) and *s(LnL)*) appear highly variable without any clear geographical pattern. Comparing quinoa samples through time at Antofagasta de la Sierra (hereafter: Antofagasta) shows a trend towards lower allelic diversity (*N*_*all-rar*_) and expected heterozygosity (*H*_*e*_) in the modern sample (#12) compared to the ancient ones (#13–15,17–19). Selfing rate (*s(LnL)*) increased significantly (P<0.05) from the most ancient samples (#17–18) to the intermediate (#13–14) and modern ones (#12).

### Genetic structure in time and space

The discriminant analysis reveals a neat distinction between ancient and modern samples, ancient samples showing little affinity to modern samples, particularly for the geographically closer sample #12 (Fig 1B, Table F in S1 Appendix). Among modern samples, genetic structure reflected the geographical sampling, with most samples showing a marked identity (Fig 1B–1C, Figures D and E in S1 Appendix). Some sites showed strong affinities between them with a clear ecogeographical link (samples #1,2 are from NE humid valleys, and samples #10,11 from arid highlands) while others are more difficult to interpret in simple ecogeographical terms (sample #8, from a mid-altitude mesothermic valley, shows strong affinity to sample #7, from cold and arid highlands).

Among ancient samples, genetic structure is associated to the age of the seeds. The two main clusters identified separate the more recent samples (#13–15) from the older samples (#17–19) (Fig 1B, Figures D and E in S1 Appendix). Affinities of sample #16 are unclear as it is composed of only two seeds with very different genotypes and from a geographically distant location. Although collected from residential or storage places, or from ritual deposits, all likely to have received seeds from various fields or sites, the ancient samples (#13–19) each showed a fairly high homogeneity, similar to that of modern samples collected in separate fields. Samples #13–15 (*ca* 690–796 cal BP) grouped together in spite of differences in seed color (#13,14 are white, #15 is dark). The two seeds from sample #16 (*ca* 1270 cal BP) were assigned to this same group. Samples #17 and 18 (*ca* 1364 cal BP) were assigned to a distinct group, which also included the oldest sample #19 (*ca* 1796 cal BP), being differently colored (#17,19 are white, #18 is dark). Interestingly, additional structure analysis separates dark-seeded quinoa samples (#15,18) and white-seeded samples from the same age and locality (#14,17 respectively) (Figure E in S1 Appendix, k = 12). Yet the wild-form samples remain genetically close to the white-seeded samples of their respective time x location set.

### Inference of local demographic history

**Fig 2:**
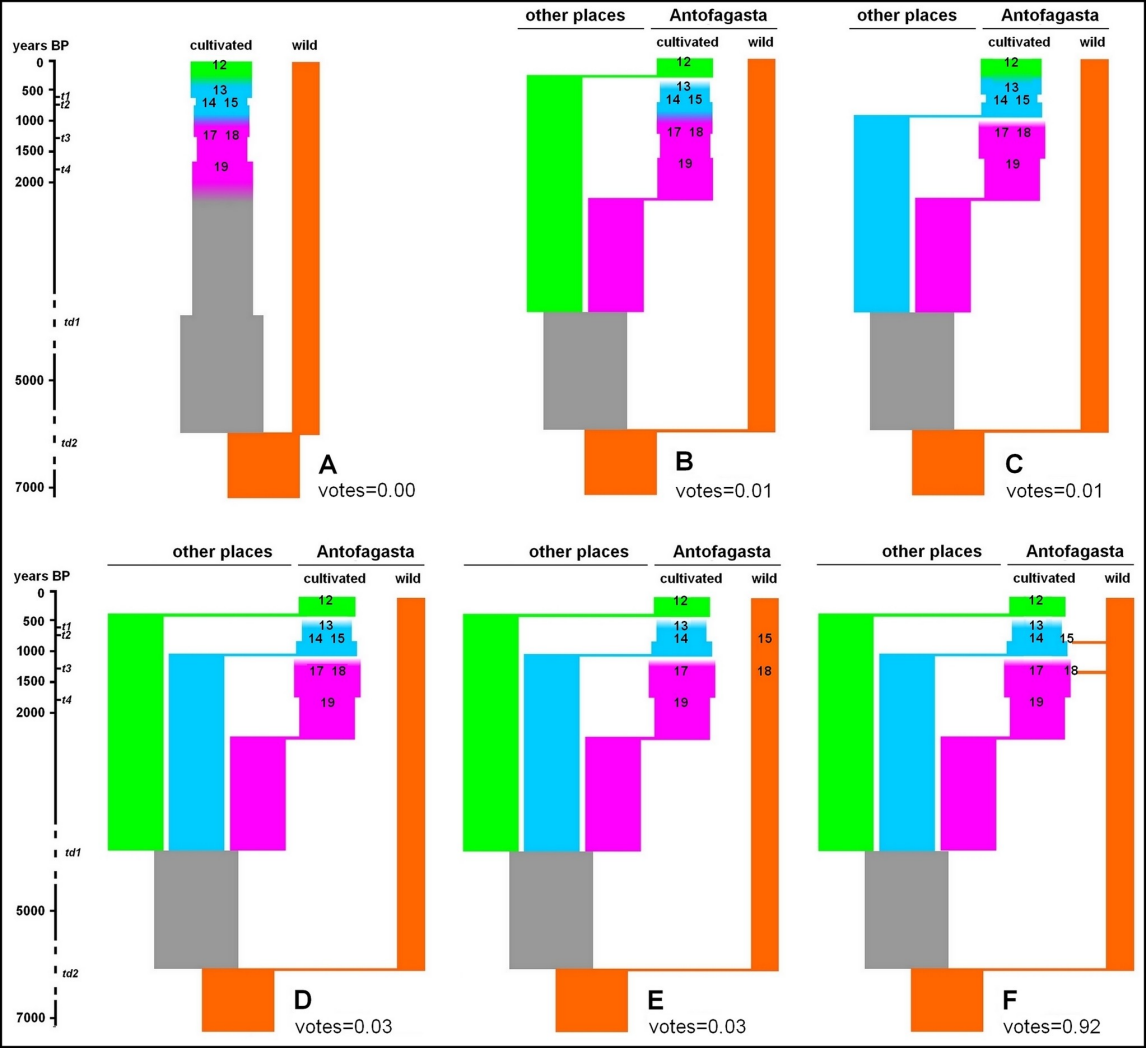
Graphical representation of alternative genetic relationships between modern and ancient quinoas from Antofagasta. The coalescence-based modeling examined six scenarios: (*A*) direct chronological filiation between all samples, mixing cultivated and wild forms, (*B*) replacement of all ancient quinoas by modern quinoas, (*C*) filiation between modern quinoas and intermediate ancient quinoas, both replacing the oldest quinoas, (*D*) successive replacement of the three groups of quinoas: modern, intermediate, ancient, (*E*) same scenario as previously but differentiating between cultivated and wild forms, (*F*) same scenario as previously but with admixture between cultivated and wild forms. Numbers in bold refer to modern (#12) and ancient quinoa samples (#13–15,17–19) described in Table B in S1 Appendix. Vertical axis shows years BP with present at the top. Proportion of votes received from the random forest classification of the observed data is shown for each model. *t*_*n*_ and *td*_*n*_ are time parameters used in the approximate Bayesian computation analysis (see Table G in S1 Appendix).

At Antofagasta, one modern sample and six ancient samples offer a temporal series covering almost two millennia. Analyses of genetic structure of these samples reveal three main genetic groups: modern (#12), intermediate (#13–15), and ancient (#17–19) (Fig 1C). The differentiation among them could be due to genetic drift between sampling times within a single population or to divergence among distinct populations that locally arrive at different times, replacing the preexisting genetic pool. In order to distinguish between these two alternative processes we built demographic scenarios in which the two temporal and genetic discontinuities are either simulated as genetic drift within a single population (Fig 2A), or mixtures of drift and replacement (Fig 2B–2D). As dark seed quinoa samples (#15,18) show some differentiation from white seeds (see Figure D for k = 14 and Figure E for k = 9 or higher in S1 Appendix), two additional scenarios were tested: one differentiating between cultivated and wild compartments (Fig 2E), the other considering an admixture event between both compartments (Fig 2F).

The coalescence analysis clearly identifies as the model with the best fit (votes = 0.92, posterior probability = 0.99) the demographic scenario in which three genetic clusters belong to three separate cultivated gene pools, with gene flow from a wild compartment producing dark seeds (Fig 2F). We estimated effective population sizes for the 3 quinoa samples around few 100 individuals and around 30 individuals for the wild gene pool (Table G in S1 Appendix). Admixture proportions from the wild pool of dark seeds was high but lower than 0.5 (point estimates and 95% highest posterior density intervals are reported in Table G in S1 Appendix). Posterior probability distributions for other parameters of the model (*e*.*g*. ancestral population effective size, time of divergence) were indistinguishable from priors (Table G in S1 Appendix).

## Discussion

Using a coalescent-based approach, this study provides the first evidence of a significant change in the demographic history of quinoa in the Andes over 18 centuries. The analysis of modern and ancient samples from Antofagasta reveals that genetic differentiation among samples from different times cannot be explained by genetic drift. Instead, the most likely scenario in this locality is the replacement of preexisting quinoa gene pools with new exogenous gene pool. This process occurred at least twice in the last 18 centuries: first, between 1364 and 796 cal BP, well before the Inka and Spanish conquests—respectively initiated 568 and 483 years ago in Northwest Argentina [31]—, and then between 690 cal BP and today, an interval of time covering the Inka, Colonial and Republican periods. The general assumption of successive genetic bottlenecks for the Andean quinoa—related to the initial events of hybridization and domestication, and then to the Spanish Conquest [32]—thus does not seem to apply at a local scale. This should be tracked back now over a larger geographic area, particularly the Central Andes where less extreme climatic conditions and a distinct socio-historical context might have led to other patterns of genetic change in quinoa. In the dry Andes, these two events of gene pool replacement appeared associated with quite different socio-environmental dynamics, namely: a phase of *agricultural intensification* initiated 1100 years ago followed by an opposite phase of *farming marginalization* in the Colonial and Republican periods.

### Intensification of agriculture

Intensification of agriculture by local societies starting 1100 BP was contemporary to the increasing aridity, which reduced water availability for crops and pastures in the region [13,17]. Large irrigation infrastructures were then established, probably associated with an increase in population density [29]. Although rare in the region, these intensified crop-pasture systems allowed to expand the productive land area from small humid river banks to broader alluvial terraces [33]. Adaptation of quinoa to these new climate and farming conditions could have occurred in two ways: either by local selection for ever more drought-tolerant variants or variants apt for new intensified fields, or directly by replacing local varieties with new ones from other regions. Our model of quinoa demographic history in Antofagasta showing the introduction of a new gene pool in the 1364–796 BP period (Fig 2F) is congruent with the second hypothesis, and concurs with the intense interregional connections at that time [29]. The grouping of samples #13–15 from Antofagasta with sample #16 from mesothermal valleys (Fig 1B–1C) suggests that the same gene pool might have circulated between dry highlands and valleys in the 1270–690 BP period.

As reported in other Andean regions [34], modern weedy (dark-seeded) quinoa appeared genetically related to sympatric cultivated (white-seeded) quinoa populations. Black chenopod seeds are generally assigned to the weed sub-species *Ch*. *quinoa* ssp. *melanospermum* and their relative frequency in archaeological remains is indicative of the degree of seed selection by past cultivators [35,36]. The presence of dark seeds (#15,18) in archaeological food processing places suggests the prolonged use of a combination of domesticated and weed chenopod grains by past populations in Northwest Argentina [37], a feature also observed elsewhere in the Americas [36,38].

Another likely cause of the changes in quinoa demographic history relates to evidence of a generalized warfare in the dry Andes in the 750–600 BP period [11,39,40], a situation exacerbated by the competition for scarce water resources [11,40], likely disturbing local seed-supply networks. Compared to the previous social system based on small villages, more complex and authority-centered societies at that time [41,42] could also have impacted on seed availability and circulation in a trend towards less diverse crop practices and genetic resources.

In this context of coincident changes in climate, crop technology and society, our estimates of effective population size (Table G in S1 Appendix) suggest that the quinoa gene pool cultivated at Antofagasta *ca* 796–690 BP (samples #13,14) had a narrower genetic base and higher selfing rates than in the previous periods (samples #17,19). As the scenario of genetic drift within a single population is rejected by the coalescent-based analyses, the lower diversity of the cultivated quinoa at Antofagasta *ca* 796–690 BP is explained more by the displacement of local varieties by introduced ones with a narrower genetic base than by alternative hypotheses of enhanced selection for new cropping systems or loss of genetic resources due to endemic political unrest. In this perspective, agricultural intensification with newly introduced varieties can be considered as a risk-buffering strategy developed by ancient Andean peoples who, like other societies in the world, sought to ensure food security in a context of rising population, political conflicts and deteriorating climate [43,44]. This observation supports the idea that social and environmental stress can stimulate cultural innovation [4,16]. The brief Inka rule at this extreme end of the Andes continued this process of agricultural intensification as suggested by the appearance of large, albeit scattered, terrace and irrigation systems in the region [17,45].

### Crop farming marginalization

Crop farming marginalization in the Andean highlands has been frequently attributed to the Spanish conquest [46–48]. Undeniably, the European intrusion affected the structure of native societies and their subsistence systems across the Andes, including local farming activities [2,49–51]. Still, in the dry Andes the new mercantilist order prioritizing mining and caravan trading remained dependent on local crop-pasture systems for its food and forage supply [52,53]. Recent studies report the continuation, after the Spanish conquest, of local crop-pasture systems and food-storage facilities which allowed native populations in the remote highlands of Norwest Argentina to preserve relative autonomy and control over natural resources during the Colonial and Republican periods [33]. Yet, these persisting crop-pasture systems stood vulnerable to climatic variations. A multidecadal drought in the 1860-1890s caused a severe mortality in the region [54], likely affecting local agriculture. Immediately after that time, socioecological changes related to emergent industrialization, urbanization, and globalization led to further rural depopulation and cropland abandonment throughout the 20th century [18]. Under these cumulative factors, the relatively intensified crop-pasture systems built up during the pre-Hispanic period—and partly maintained until the 19th century—were dismantled in the study area and local agriculture returned to small-scale cropping and extensive pastoralism. In some southern highlands, intensified agricultural fields could have fallen into disuse much earlier—late 18th century or earlier—due to an emphasis on animal husbandry, whereas crop production continued as an important activity in the neighboring mesothermal valleys [50]. We found that in the Colonial and Republican periods, a second event of gene pool replacement occurred in the quinoa cultivated at Antofagasta, which resulted in local quinoa gene pools of lower allelic diversity (Table E in S1 Appendix). As shown by population genetics theory [55], such a process of local gene pool replacement does not necessarily imply a loss of genetic diversity through time at the metapopulation scale. It does, however, support the view that gene pool replacement linked to social and environmental changes can result from opposite trajectories of agricultural intensification or marginalization. Such historical shifts in farming activities are characteristic of agriculture in extreme environments [37,56,57] and not only in remote times [58,59].

## Material and methods

See S1 Appendix for an extended version of the methods.

### Seed sample collection, archaeological material and datings

Ancient and modern quinoa seed samples were collected from the sites described in Fig 1A and Table B in S1 Appendix. Archaeologists collected intact, non-charred samples of ancient quinoa in five sites related to agro-pastoralist societies from Northwest Argentina, covering the time span 1796–690 BP (detailed description in Section A in S1 Appendix). Four of the archaeological sites are located in arid highlands near the town of Antofagasta de la Sierra (Catamarca province): Cueva Salamanca 1 (sample #19; [60]), Punta de la Peña 9 (samples #17,18), Punta de la Peña E (samples #14,15), and Punta de la Peña 4 (sample #13; [61]). The fifth site, Cueva de los Corrales 1 (sample #16; [62]) corresponds to an area of mesothermal valleys in the Tucuman province. Sedimentary samples containing exceptionally preserved ancient quinoa remains from these sites were submitted to laboratory separation and concentration techniques (dry sieving and picking under magnifying glass) shortly before AMS dating and molecular analysis of seeds. In 2006–2007, an independent research team collected modern quinoa seed samples from 12 sites representative of different environments in the Andean highlands and valleys of Argentina [26,30]. Ancient and modern quinoa seeds were not in contact during their sampling, storage and manipulations.

### DNA extraction, microsatellite genotyping, and genetic data analysis

We extracted DNA from 81 modern and 144 ancient quinoa seeds according to the procedures described in Section C in S1 Appendix. DNA extraction was successful for all the modern seeds, while we recovered well-preserved DNA from only 76 ancient seeds (53%). To avoid contamination between ancient and modern DNA, we rigorously separated in time and space all the DNA extraction, DNA quality control and microsatellite amplification procedures detailed in Section C in S1 Appendix. We started by working on the ancient archaeological quinoas in a specific laboratory dedicated to ancient DNA. Once all the extractions and amplifications of ancient quinoa seeds were completed, we then proceeded to the extraction and amplification of modern quinoas in a distant laboratory, without any spatial connection with the previous one.

Ancient and modern quinoas were genotyped using 24 microsatellite loci (Table C in S1 Appendix). We did not find an ancient genotype identical to any other ancient or modern genotype, which proves the absence of contamination (see Section C in S1 Appendix). Descriptive genetic diversity (allelic richness, heterozygosity), inbreeding fixation coefficient (*F*_IS_) and genetic differentiation (*F*_ST_) were calculated in R using the packages *adegenet* and *hierfstat* [63,64]. The number of multilocus genotypes (*MLGs*) was computed in R using the package *poppr* [65]. Diversity indexes were standardized using a rarefaction approach in ADZE [66]. We estimated selfing rates for each sample in two independent ways, either from *F*_IS_, or using the maximum likelihood approach implemented in RMES (see Section C in S1 Appendix). The genetic structure of the samples was examined using the program STRUCTURE [67], principal component analysis (PCA), and discriminant analysis of principal components (DAPC) using *adegenet*. An approximate Bayesian computation approach using random forests (ABC-RF) [68,69] was used to evaluate alternative models of demographic history of quinoa found around Antofagasta where one modern (#12) and six ancient seed samples (#13–15,17–19) offer a temporal series covering 18 centuries. A classification vote system, which represents the frequency of each alternative model in the collection of classification trees, identified the model best suited to the observed dataset [68].

## Supporting information

S1 Appendix Section A. ARCHAEOLOGICAL SITE DESCRIPTION
Table A. Succinct chronology of climatic and social changes in the Southern dry Andes.
Section B. SEED SAMPLE DESCRIPTION
Table B. Seed sample description.
Section C. DNA EXTRACTION, SIMPLE SEQUENCE REPEAT GENOTYPING, ANCIENT DNA QUALITY CONTROL, AND POPULATION GENETIC ANALYSIS
Table C. Microsatellite loci, allele expected size, observed size range, allele number, and missing data points in modern (n = 81) and ancient (n = 76 successfully genotyped out of 144) quinoa seed samples.
Table D. Percentage of missing data per successfully genotyped quinoa seed sample, calculated across 24 microsatellite loci.
Table E. Diversity and selfing rates in the 19 studied quinoa seed samples.
Table F. Pairwise FST values between samples.
Table G. Prior and posterior probability distribution of model parameters for the approximate Bayesian computation analysis.
Figure A. Sequence alignment from QAAT024 locus alleles of 4 modern (EST and CHEN458 samples) and 4 ancient (AP9 and PPE samples) quinoa genotypes.
Figure B. Sequence alignment from QAAT087 locus alleles of one modern (CHEN 420) and three ancient (PPE, ACS, AP9) genotypes.
Figure C. Inference of number of clusters in DAPC.
Figure D. Individual assignment probability to each group from Discriminant Analysis of Principal Components (DAPC).
Figure E. Individual assignment probability to each group from STRUCTURE.
Figure F. First two principal components for the Principal Component Analysis (PCA)
(PDF)

S1 File SEED SAMPLE ALLELIC PROFILES (ReadMe). S1–File-WINKEL-et-al-Allelic-profiles-ReadMe.
(PDF)

S2 File SEED SAMPLE ALLELIC PROFILES. (Dataset). S2–File-WINKEL-et-al-Allelic-profiles-Dataset-(2018-04-05).
(CSV)
