# Supplementary material for: Discontinuities in quinoa biodiversity in the dry Andes: An 18-century perspective based on allelic genotyping
Source: PLoS One. 2018 Dec 5;13(12):e0207519. doi: 10.1371/journal.pone.0207519 (PMC6281180; doi:10.1371/journal.pone.0207519)
Supplement: S1 Appendix — Section A. ARCHAEOLOGICAL SITE DESCRIPTION Table A. Succinct chronology of climatic and social changes in the Southern dry Andes. Section B. SEED SAMPLE DESCRIPTION Table B. Seed sample description. Section C. DNA EXTRACTION, SIMPLE SEQUENCE REPEAT GENOTYPING, ANCIENT DNA QUALITY CONTROL, AND POPULATION GENETIC ANALYSIS Table C. Microsatellite loci, allele expected size, observed size range, allele number, and missing data points in modern (n = 81) and ancient (n = 76 successfully genotyped out of 144) quinoa seed samples. Table D. Percentage of missing data per successfully genotyped quinoa seed sample, calculated across 24 microsatellite loci. Table E. Diversity and selfing rates in the 19 studied quinoa seed samples. Table F. Pairwise FST values between samples. Table G. Prior and posterior probability distribution of model parameters for the approximate Bayesian computation analysis. Figure A. Sequence alignment from QAAT024 locus alleles of 4 modern (EST and CHEN458 samples) and 4 ancient (AP9 and PPE samples) quinoa genotypes. Figure B. Sequence alignment from QAAT087 locus alleles of one modern (CHEN 420) and three ancient (PPE, ACS, AP9) genotypes. Figure C. Inference of number of clusters in DAPC. Figure D. Individual assignment probability to each group from Discriminant Analysis of Principal Components (DAPC). Figure E. Individual assignment probability to each group from STRUCTURE. Figure F. First two principal components for the Principal Component Analysis (PCA) (PDF) [file pone.0207519.s001.pdf]

WINKEL T. et al. Discontinuities in quinoa biodiversity in the dry Andes: an 18-century perspective based on allelic genotyping

S1 APPENDIX

Section A. ARCHAEOLOGICAL SITE DESCRIPTION

Four of the sites analyzed in this study are located in a dry and cold highland environment (*puna*) between 3600 and 3700 masl. They belong to the Punilla River basin in the Catamarca province (Argentina). The fifth site, Cueva de los Corrales 1, corresponds to an area of mesothermal valleys at 3000 masl in the Tucuman province. We present here the sites in the chronological order, starting from the most recent one.

The site **Punta de la Peña 4 (sample #13: AP4)** is a rock-shelter with a large protected area that occurs in the upper portion of the ignimbrite cliff of Punta de la Peña. Its archaeological occupation dates back to *ca* 9000 Before Present (BP). The layers 1 to 3 dated between *ca* 760 and 460 cal BP, with a sandy-silty sedimentary matrix. A high density and diversity of archaeological remains in the form of artifacts, ecofacts and structures with well-preserved plant and faunal remains characterized these layers. Within layer 3, the 3x lens was composed of a pit fire associated to numerous intact (dried) or charred quinoa seeds dated *ca* 690 ± 50 cal BP (UGA-15090, cal. 2σ, 95.4%: 1228-1398 Common Era (CE)) [61]. Other quinoa seeds come from a carbonaceous dispersion in lens 3z, 700 ± 40 cal BP (UGA-15089, cal. 2σ, 95.4%: 1249-1392 CE) and non-carbon sectors of this same lens dated 760 ± 40 cal BP (UGA-15089, cal. 2σ, 95.4%: 1190-1294 CE).

The site **Punta de la Peña E (samples #14,15: PPE-w, PPE-d)** corresponds to an archaeological assemblage interpreted as an intentional deposit due to propitiatory practices associated with fertility. It is composed of a ceramic vessel containing a folded textile fragment, covered with a filling of clay sediment of intense *reddish* color and a high volume of plant material. From this sedimentary filling come numerous intact quinoa seeds dated *ca* 796 ± 24 cal BP (AA-105653, cal. 2σ, 95.4%: 1206-1275 CE). The location of the deposit corresponds to a horizontally raised platform on top of the ignimbrite cliff of Punta de la Peña, at the foot of which there are numerous residential and productive spaces covering a wide temporal sequence.

East of the Puna, **Cueva de Los Corrales 1 site (sample #16: TC2)** is a cave located at 2966 masl in an area of mesothermal valleys, on the west bank of the lower Los Corrales river (El Infiernillo,

Tucumán). It comprises a stratigraphic sequence 30 cm thick made of two layers of anthropic origin, separated into three extractions in each case: Layer 1 (1st, 2nd and 3rd extractions) and Layer 2 (1st, 2nd and 3rd extractions). The excellent natural conditions of preservation allowed the recovery of a wide variety of archaeological remains of both inorganic and organic origin. Cueva de Los Corrales 1 has been defined as a multiple activity site with emphasis on processing, consumption and disposal of animal and plant food resources [62]. Quinoa seeds analyzed in this paper come from Layer 2 (1st and 2nd extractions) dated *ca* 1270 ± 30 cal BP (UGA-22266, cal. 2σ, 95.4%: 663-859 CE).

The Alero 1 in the **Punta de la Peña 9.I site (samples #17, 18: AP9-w, AP9-d)** is located on the edge of the plateau that defines the Sector I of the site, close to a number of stone-walled structures corresponding to agro-pastoralist occupations between *ca* 1500 and 1100 BP. The rocky repair consists of large blocks detached from the ignimbrite cliff of Punta de la Peña. It is apparently collapsed and its walls and top are sooted. The context of interest for this work (Layer 2) lies below a sandy layer (Layer 1), possibly of eolian origin. Layer 2 is composed of a sandy matrix with a wide variety of plant remains recovered in high concentration, together with faunal remains, cordage and scarce lithic and ceramic materials. Macrobotanical remains of *Chenopodium* include dried, non-charred quinoa seeds and fragments of stems and distal ends of the panicles. Quinoa seeds were dated *ca* 1364 ± 20 cal BP (AA-107154, cal. 2σ, 95.4%: 655-766 CE). There are also charred quinoa seeds that constitute waste material from post-harvesting and processing activities, probably for culinary purposes.

Cueva Salamanca 1 (sample #19: ACS) is a large cave on the northern margin of the Las Pitas River at an elevation of 3665 masl. The cave is 11 m wide, 8 m deep and 7 m tall (77 m^2). A total of 30 m^2 of the site has been excavated so far. Three stone structures are found beside the back wall. The stratigraphy of the cave covers at least 5000 years, and the sediments are the result of natural processes–mostly eolian–and human activity. A series of ten living surfaces that contain hearths, tools, lithic debitage, vegetal remains and grass features have been excavated. Of interest for this paper is the upper stratum, which overlays a lens of volcanic ash. A radiocarbon date *ca* 4500 BP immediately below the volcanic ash gives a *terminus post quem* for the volcanic episode and thus the upper stratum [60]. This stratum–level 1(2ª)–included two hearths, abundant lanceolate nonstemmed obsidian points, a stemmed point of the Punta de la Peña C type [70], grinding stones, quinoa seeds dated *ca* 1796 ± 93 cal BP (AA-107153, cal. 2σ, 95.4%: 231-358 CE), quinoa stems dated *ca* 1742 ± 22 cal BP (AA-107155, cal. 2σ, 95.4%: 250-409 CE), and a spherical pit-like feature that cut through the volcanic ash lens. Above is another stratigraphic unit–level 1(1ª)–that consists of a loose sandy surface that included ceramic sherds, a small lanceolate and nonstemmed projectile point

attributed to the Peñas Chicas E morphological type [70] and three sub-circular stone features that lacked anthropogenic content.

Table A. Succinct chronology of climatic and social changes in the Southern dry Andes.

Years BP	Years CE	Climate	Society	Quinoa samples
127 to the present	1890 to the present	global warming dry phase	continued rural emigration in a context of accelerated technological change, industrialization, urbanization	#1-12
157 to 127	1860 to 1890	extreme drought	rural depopulation due to mortality and emigration	
370 to 157	17th to 19th centuries	dry phase	efficient crop-pasture systems sustaining regional economy, abandonment of intensified crop fields in Southern highlands due to an emphasis in animal husbandry	
482	1535	dry phase	beginning of Spanish Conquest facing a century of local rebellion	
567 to 482	1450 to 1535	dry phase	Inka colonization, continued agricultural intensification	
1100 to 567	850 to 1450	dry phase	localized agricultural intensification (irrigation, terracing), corporate societies	#13, 14, 15
1500 to 1100	450 to 850	dry phase	agropastoralist societies	#16, 17, 18
5000 to 1500	-3000 to 450	humid phase	pastoralism, early plant domesticates	#19
8000 to 5000	-6000 to -3000	dry phase	hunter-gatherers	
12000 to 8000	-10000 to -6000	humid phase	hunter-gatherers	

Section B. SEED SAMPLE DESCRIPTION

Table B. Seed sample description.

N°	Code	Site, Department, Province[a]	Longitude, latitude[b]	Altitude m.a.s.l	Seed count /color[c]	Age cal BP[d]
1	CHEN 458	Morro de Pucará, Sta Victoria, Salta	-64.97, -22.18	2645	8/w	modern
2	CHEN 461	Poscaya, Sta Victoria, Salta	-65.08, -22.45	3208	8/w	modern
3	CHEN 466	S. José del Aguilar, Sta Victoria, Salta	-65.17, -22.34	3960	8/w	modern
4	CHEN 446	Humahuaca, Jujuy	-65.18, -23.08	3823	7/w	modern
5	CHEN 275	1485 Coctaca, Humahuaca, Jujuy	-65.28, -23.15	3215	8/w	modern
6	CHEN 414	La Poma, Salta	-66.20, -24.72	3016	3/w	modern
7	CHEN 272	1482 La Poma, Jujuy	-65.82, -23.85	3480	8/d	modern
8	EST	Las Estancias, Andalgalá, CTM	-66.03, -27.58	1650	8/w	modern
9	CHEN 427	Puesto Sey, Susques, Jujuy	-66.48, -23.95	4012	8/w	modern
10	BL	Barranca Larga, Belén, CTM	-66,74, -26.98	2400	8/w	modern
11	CHEN 420	Antofallita, Los Andes, Salta	-67.52, -25.25	3498	5/w	modern
12	ANT	Antofagasta de la Sierra, CTM	-67.42, -26.05	3590	8/w	modern
13	AP4	Punta de la Peña 4, ADLS, CTM	-67.33, -26.02	3590	10/w	690 ± 50
14	PPE-w	Punta de la Peña E, ADLS, CTM	-67.33, -26.02	3590	19/w	796 ± 24
15	PPE-d	Punta de la Peña E, ADLS, CTM	-67.33, -26.02	3590	10/d	796 ± 24
16	TC2	Cueva Corrales 1, Tafí del Valle, TUC	-65.80, -26.73	2966	2/w	1270 ± 30
17	AP9-w	Punta de la Peña 9, ADLS, CTM	-67.33, -26.02	3590	25/w	1364 ± 20
18	AP9-d	Punta de la Peña 9, ADLS, CTM	-67.33, -26.02	3590	8/d	1364 ± 20
19	ACS	Cueva Salamanca 1, ADLS, CTM	-67.33, -26.01	3665	2/w	1796 ± 23

[a] ADLS: Antofagasta de la Sierra, CTM: Catamarca, TUC: Tucumán;

[b] longitude and latitude in decimal degree values;

[c] seed count in sample / seed color is dark (d) or white (w);

[d] modern seed samples were collected in 2006-2007; ancient seed samples were dated using the AMS radiocarbon dating method with "cal BP" meaning: calibrated years before present, and "present" referring to year 1950 CE (dating sources for each sample are detailed in **Section A**).

Section C. DNA EXTRACTION, SIMPLE SEQUENCE REPEAT GENOTYPING, ANCIENT DNA QUALITY CONTROL, AND POPULATION GENETIC ANALYSIS

Prevention of contamination. Following recommendations to minimize the risk of exogenous DNA contamination and ensure the reliability of the results [71], dissections, extractions and pre-PCR processing of ancient and modern seeds were rigorously separated in time and space. We first processed ancient seeds in a specific laboratory dedicated to ancient DNA under sterile conditions. In the ancient DNA laboratory, we purchased and used new consumables and extraction kits, with the room cleaned and exposed to UV overnight after each DNA extraction cycle, in order to destroy possible traces of DNA between successive extractions. We wore protective clothing and footwear. Once all the extractions and amplifications of archaeological seeds were completed, we then proceeded to the extraction and amplification of modern seeds in a distant laboratory, without any spatial connection with the previous one.

Genotyping. We worked on 81 modern and 144 ancient quinoa seeds for DNA extraction according to the below-described procedures. In modern as well as ancient seeds, we dissected seeds under a stereomicroscope (Leica MZ 16, Leica camera DFC 280) to separate the embryo from the central perisperm. We next extracted total DNA from embryos. Quinoa embryos (*ca* 1-3 mm length, 1 mm thick) were dissected one seed after the other, using sterile dissection equipment and binoculars. DNA extraction was successful for all the modern seeds, while we recovered well-preserved DNA from only 76 ancient seeds (53%). This level of ancient DNA recovery reflects the high preservation of genetic material in dry environments as pointed out by [72], conditions still improved in the dry Andes by cold temperatures and oxygen scarcity at high altitude [73,74]. Despite these favorable conditions, there appears to be a time limit for the preservation of quinoa seeds in archaeological contexts [75].

DNA extraction. Total DNA extraction was obtained using DNeasy Plant Kit (Qiagen, Hilden, Germany) following the DNeasy Tissue Kit Handbook protocol with two 50 µL final elution. We extracted DNA by sets of no more than 12 samples per half-day, with one negative extraction control for each set of extractions.

PCR amplification. All quinoa samples were initially genotyped at 25 polymorphic microsatellite loci described by Mason et al. [76] and Jarvis et al. [77]. PCR consisted of a final volume of 10 µL containing 0.2 µM of each primer, 2 or 4 µL of DNA solution (depending on the storage quality of the sample), and 5 µL of kit multiplex PCR kit (Qiagen). The amplification parameters were 15 min of

95 °C followed by 30 cycles (40 cycles for ancient quinoa samples) of 94 °C for 60 s, 56 °C for 120 s, and 72 °C for 60 s, with a final extension step for 30 min of 60 °C in a Mastercycler epgradients (Eppendorf). We systematically performed negative controls to check for possible contamination. We independently amplified each individual two times, retaining only congruent results. Amplifications products were separated on an ABI-3100 Automated Sequencer at the SFR SEM platform and analyzed with Genemapper 4.0 (Applied Biosystem) using Genescan-500LIZ size standard (Applied) with two investigators eye checking for allele scoring. Genetic analyses included only reproducible alleles, present in both replicates, and accessions with reliable information for at least 24 of the 25 loci. We discarded locus QAAT100 because of its complex motive and size results out of range). Microsatellite loci, expected size and observed size range, number of alleles per locus and missing data are included in **Tables C** and **D**.

Table C. Microsatellite loci, allele expected size, observed size range, allele number, and missing data points in modern (n=81) and ancient (n=76 successfully genotyped out of 144) quinoa seed samples. All loci are derived from Mason et al. [76] except KGA 003 and KGA 20 derived from Jarvis et al. [77].

Locus	Expected size (bp)	Observed size (bp)	Allele number	Missing modern data points	Missing ancient data points
KGA003	150	139-174	12	1	0
KGA020	177	152-204	22	1	3
QAAT001	182	131-225	16	5	9
QAAT011	197	162-237	24	5	8
QAAT022	194	138-253	31	2	1
QAAT024	198	169-229	19	1	1
QAAT026	181	172-253	17	20	4
QAAT027	165	147-215	20	4	7
QAAT050	199	186-242	26	0	1
QAAT062	187	157-215	17	7	1
QAAT071	170	130-309	37	2	1
QAAT074	186	160-218	15	5	12
QAAT078	196	176-253	14	4	3
QAAT087	185	165-211	14	0	1
QAAT088	151	99-173	21	0	46
QAAT097	177	161-215	18	1	13
QAAT106	299	288-325	11	0	22
QAAT112	199	176-227	13	1	4
QATG064	177	167-182	5	0	4
QCA037	188	173-194	10	1	22
QCA053	189	166-200	13	3	0
QCA057	163	155-191	11	1	6
QGA003	150	125-197	25	1	0
QGA024	195	169-229	19	1	1
QAAT100	349	*out of range*	*complex motive*	*discarded*	*discarded*

Table D. Percentage of missing data per successfully genotyped quinoa seed sample, calculated across 24 microsatellite loci. (#1-12: modern, n=81; #13-19: ancient, n=76).

n°	Sample code	Number of genotypes	Missing data	% missing data
1	CHEN458	8	2	1.0
2	CHEN461	8	7	3.6
3	CHEN466	6	2	1.4
4	CHEN446	6	8	5. 6
5	CHEN275	8	8	4.2
6	CHEN414	2	3	6.2
7	CHEN272	8	19	9.9
8	EST	8	6	3.1
9	CHEN427	8	6	3.1
10	BL	7	6	3.6
11	CHEN420	4	4	4.2
12	ANT	8	4	2.1
	mean modern			*3,9 ± 2.4*
13	A-P4	10	61	25.4
14	PPE-w	19	7	1.5
15	PPE-d	10	3	1.3
16	T-C2	2	6	12.5
17	A-P9-w	25	65	10.8
18	A-P9-d	8	23	12.0
19	A-CS	2	11	22.9
	mean ancient			*12.3 ± 9.4*

Preliminary analyses of ancient DNA quality. To check for DNA quality in ancient quinoa seeds, alleles from locus QAAT024 and QAAT087 of ancient and modern samples were direct sequenced in forward and reverse sense, using the ABI Prism BigDye Terminator Cycle Sequencing Kit 3.1 in an Applied Biosystems 3500 DNA Sequencer. Reactions containing fragments of the expected size were purified by treatment with Exonuclease I and Shrimp Alkaline Phosphatase. Enzymes were added directly to the PCR product to degrade primers and dephosphorylate dNTPs that were not consumed in the reaction and could interfere with downstream sequencing. Treatment was carried out for 15 minutes at 37 °C, followed by a 15-minute incubation at 80 °C to completely inactivate both enzymes. Base assignment was made with GeneMapper V3.0 software (Applied Biosystems). Phred quality score was settled at 20 to assure 99% of base call accuracy, as a measure of the quality of the identification of the nucleobases generated by automated DNA sequencing. Sequences were aligned using CLUSTALW [78] followed by minor manual modifications. We analyzed amplification products by comparison with the public sequence databases as nucleotide using BLASTN. Following the criteria of Meyers et al. [79], a sequence was classified as a known element when retrieved with a BLAST E-value of less than 10^{-5}.

Sequence analysis revealed amplification products corresponding to what was expected for both microsatellite loci. After BLAST search, we found modern sequences to have 100% homology with *Chenopodium quinoa* clones of microsatellite sequences with E-values of virtually zero. At locus QAAT024, we selected 6 ancient and 4 modern genotypes, 2 ancient samples failed to give positive results. Finally, we obtained 8 consensus sequences, from 4 modern and 4 ancient genotypes (**Fig A**). In the microsatellite region, a six-base pair InDel was present between these modern genotypes, according to the size of the expected fragment. The ancient sequences revealed variations at a total of seven nucleotide positions, representing 97% of sequence similarity with modern sequences. Variation at nucleotide position 70 was on only one single base in one ancient genotype. The other 6 mutations discriminated between modern and ancient sample groups, as well as among ancient genotypes (nucleotide position 229), which confirms the absence of contaminating sequences.

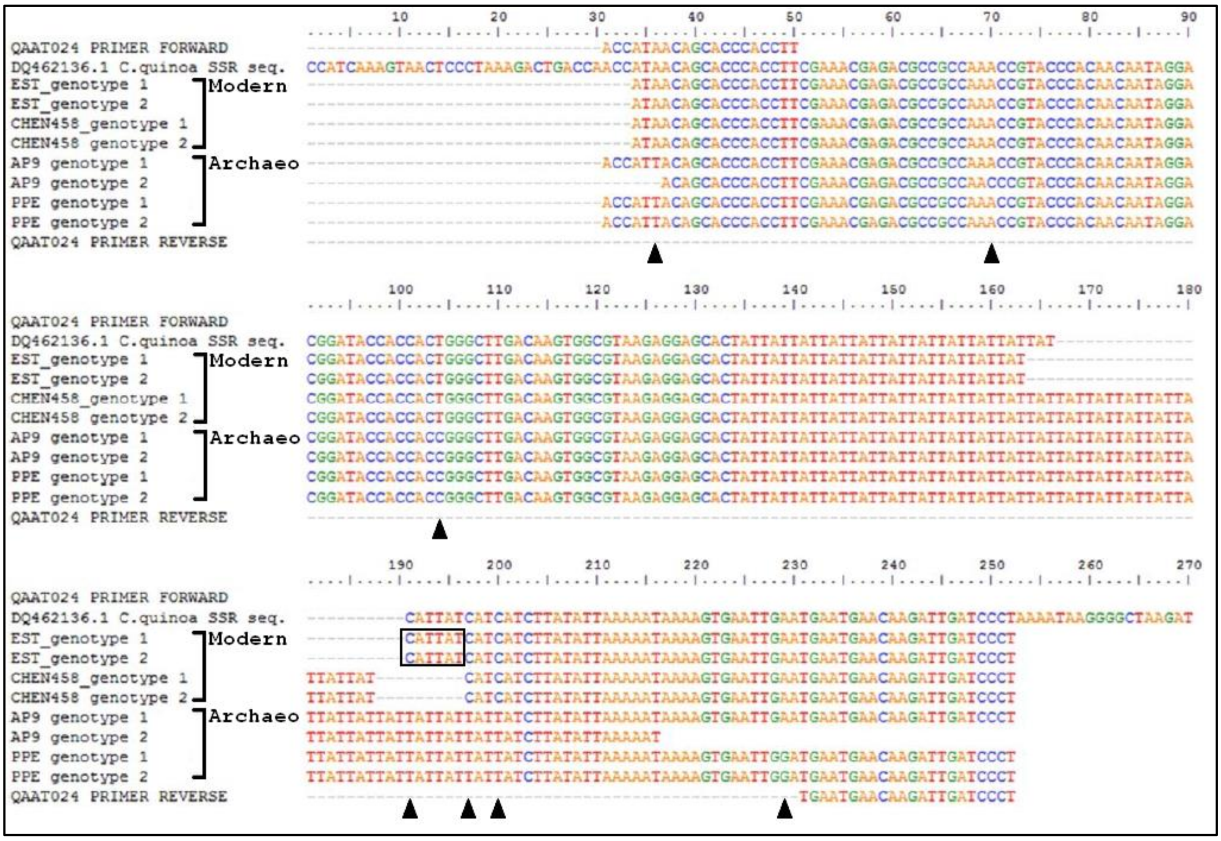

Fig A. Sequence alignment from QAAT024 locus alleles of 4 modern (EST and CHEN458 samples) and 4 ancient (AP9 and PPE samples) quinoa genotypes. Primer and NCBI accession sequences are included. Arrows show sequence variations, box shows the 6-base pair InDel.

At locus QAAT087, we obtained 4 consensus sequences, from 3 ancient and 1 modern genotype (the remaining samples failed to give positive results) (**Fig B**). Sequences from locus QAAT087 have a lower quality than locus QAAT024, not only in archeological samples but also in modern ones. Alignment analyses revealed sequence variations at a total of 7 nucleotide positions, representing 97% of sequence similarity with modern sequences.

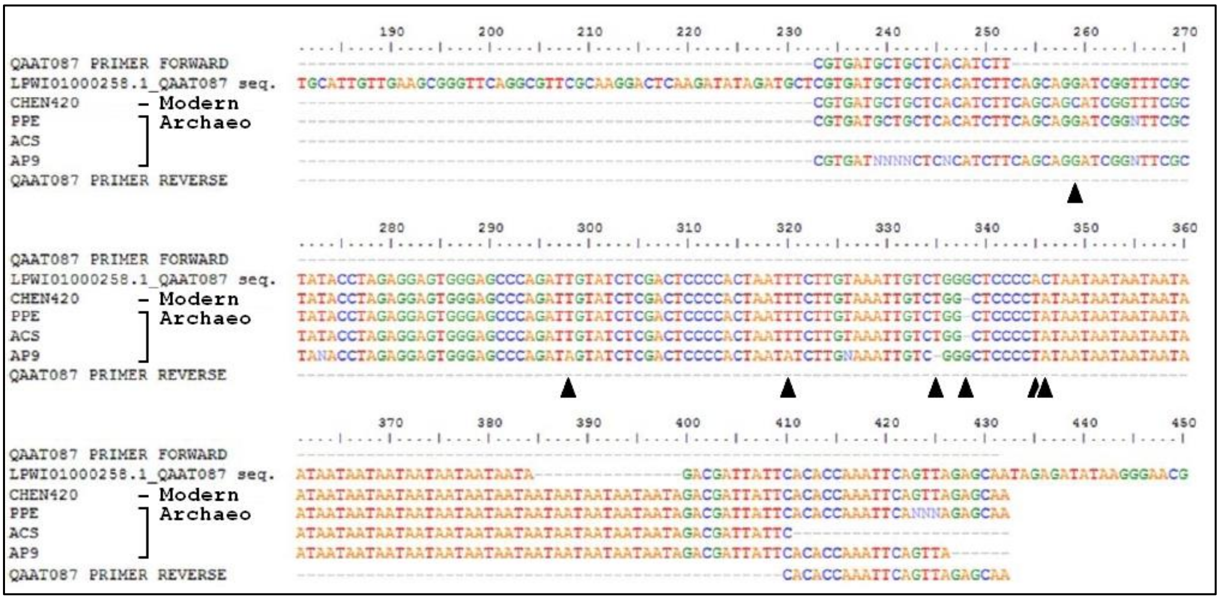

Fig B. Sequence alignment from QAAT087 locus alleles of one modern (CHEN 420) and three ancient (PPE, ACS, AP9) genotypes. Primer and NCBI accession sequences are included. Arrows show sequence variations.

Allele identities were corroborated by sequencing and CLUSTAL alignment and BLAST algorithms. Amplifications were successful for most of the samples, indicating an adequate quality of both ancient and modern DNA. Taking into account that all caution was taken to avoid contamination, our results agree with O'Donogue [72], who suggests that the microenvironment within desiccated seeds is conducive to enhanced preservation of lipids, nucleic acids and other biomolecules.

Genetic diversity indexes. We measured the genetic diversity of each sample using the allelic richness $N_{all\text{-}rar}$ [80] and the expected heterozygosity H_e. In predominantly selfing populations, we expect a strong deviation compared to Hardy-Weinberg expectations. We estimated the inbreeding fixation coefficient F_{IS} for each sample and F_{ST} for each pair of samples according to Weir & Cockerham [81]. F_{IS} and F_{ST} measure genetic differentiation within and among populations respectively; both coefficients range from zero (no differentiation) to one (complete differentiation). Analyses were performed in R using the packages *adegenet* and *hierfstat* [63,64] and the program ADZE for rarefaction analyses [66].

We expect quinoa populations to be highly selfing and therefore to display a limited number of repeated multilocus genotypes (thereafter MLG). We used the package *poppr* to count the number of MLGs present in each population (*nbMLG*) [65]. Populations with no more than two individuals sampled were removed from this analysis. We then used the rarefaction method (ADZE)

to estimate the expected MLG richness (*eMLG*) corrected for the differences in sample size. Finally, within each population, the composition in MLGs can either be balanced, or highly biased with one predominant MLG. We measured this using the Simpson diversity index (λ) that is equivalent to a multilocus expected heterozygosity.

Selfing rate estimation. Two independent estimates of selfing rates were calculated: either directly as $s(F_{IS})=2\ F_{IS}/(1+F_{IS})$ [82], or as *s(LnL)* using the program RMES (robust multilocus estimate of selfing), based on the distribution of multilocus heterozygosity, which allowed calculating a confidence interval at P=95% [83]. We used the maximum likelihood estimation with a_precision of 0.00001, a maximum number of generations of selfing set to 10 and 100000 iterations. In some populations, the high degree of homozygosity (#1,3,6) or the low sample size (samples #10,11,16,19) prevented the estimation of *s(LnL)*. Results of allelic diversity, heterozygosity and selfing rates in the 19 studied quinoa samples are shown in **Table E**.

Table E. Diversity and selfing rates in the 19 studied quinoa seed samples. (samples #1-12: modern; #13-19: ancient).

n°	*sample*	*N*	$N_{all\text{-}rar}$	H_e	*nbMLG*	*eMLG*	*λ*	F_{IS}	$s(F_{IS})$	*s(LnL)*	*CI (95%)*
1	CHEN458	8	1.00	0.02	3	2	0.406	1.00	1.00	-	-
2	CHEN461	8	1.94	0.39	8	4	0.875	0.50	0.67	0.52	0.31 - 0.71
3	CHEN466	6	1.10	0.07	5	3.6	0.778	0.80	0.89	-	-
4	CHEN446	6	1.39	0.18	6	4	0.833	0.60	0.75	0.89	0.82 - 0.94
5	CHEN275	8	2.39	0.56	8	4	0.875	0.15	0.26	0.21	0.12 - 0.37
6	CHEN414	2	-	0.05	-	-	-	1.00	1.00	-	-
7	CHEN272	8	3.05	0.70	8	4	0.875	0.02	0.04	0.12	0.03 - 0.24
8	EST	8	2.51	0.61	8	4	0.875	0.81	0.90	0.42	0.01 - 0.73
9	CHEN427	8	2.76	0.67	8	4	0.875	0.58	0.73	0.72	0.68 - 0.79
10	BL	7	1.44	0.17	7	4	0.857	0.51	0.68	-	-
11	CHEN420	4	1.36	0.17	4	4	0.750	0.82	0.90	-	-
12	ANT	8	1.81	0.36	8	4	0.875	0.28	0.44	0.69	0.65 - 0.77
13	AP4	10	2.94	0.71	10	4	0.900	0.66	0.80	0.75	0.68 - 0.82
14	PPE-w	19	2.69	0.61	19	4	0.947	0.66	0.80	0.76	0.73 - 0.80
15	PPE-d	10	2.77	0.66	10	4	0.900	0.54	0.70	0.78	0.75 - 0.82
16	TC2	2	-	0.95	-	-	-	0.97	0.98	-	-
17	AP9-w	25	3.05	0.72	25	4	0.960	0.48	0.65	0.54	0.45 - 0.61
18	AP9-d	8	3.11	0.74	8	4	0.875	0.35	0.52	0.59	0.55 - 0.69
19	ACS	2	-	0.70	-	-	-	0.36	0.53	-	-

N: number of individuals; $N_{all\text{-}rar}$: allelic richness estimated using ADZE; H_e: expected heterozygosity; *nbMLG*: number of multilocus genotypes observed; *eMLG*: MLG richness estimated by a rarefaction method; *λ*: Simpson diversity index; F_{IS}: inbreeding fixation coefficient; $s(F_{IS})$: selfing rate estimated from the F_{IS} ; *s(LnL)*: selfing rate estimated by RMES ; *CI (95%)*: confidence interval of *s(LnL) at P=95%*.

Table F. Pairwise F_{ST} values between samples. (samples #1-12: modern; #13-19: ancient).

n°	1	2	3	4	5	6	7	8	9	10	11	12	13	14	15	16	17	18
2	0.57																	
3	0.96	0.74																
4	0.91	0.66	0.81															
5	0.68	0.47	0.64	0.59														
6	0.97	0.60	0.93	0.83	0.42													
7	0.62	0.41	0.52	0.42	0.29	0.45												
8	0.67	0.48	0.57	0.50	0.35	0.46	0.18											
9	0.61	0.42	0.55	0.52	0.29	0.41	0.22	0.25										
10	0.91	0.70	0.86	0.80	0.57	0.84	0.48	0.49	0.48									
11	0.93	0.67	0.87	0.79	0.53	0.85	0.42	0.45	0.44	0.06								
12	0.78	0.58	0.72	0.54	0.50	0.68	0.35	0.43	0.44	0.69	0.67							
13	0.60	0.37	0.57	0.51	0.29	0.40	0.22	0.28	0.21	0.49	0.43	0.42						
14	0.57	0.43	0.54	0.50	0.35	0.46	0.30	0.36	0.28	0.49	0.45	0.46	0.03					
15	0.60	0.40	0.55	0.50	0.31	0.41	0.25	0.32	0.24	0.49	0.45	0.45	0.08	0.15				
16	0.85	0.47	0.76	0.62	0.35	0.49	0.26	0.24	0.21	0.67	0.60	0.52	0.12	0.22	0.13			
17	0.47	0.33	0.45	0.42	0.25	0.36	0.18	0.23	0.19	0.38	0.34	0.35	0.17	0.22	0.21	0.20		
18	0.60	0.38	0.55	0.49	0.26	0.38	0.20	0.25	0.20	0.45	0.40	0.41	0.19	0.25	0.21	0.20	0.13	
19	0.85	0.45	0.76	0.64	0.30	0.55	0.22	0.24	0.16	0.62	0.58	0.51	0.13	0.18	0.16	0.05	0.05	0.12

Genetic structure. We investigated the genetic structure among the samples with a multivariate approach, the Discriminant analysis of Principal Components (DAPC) implemented in the package *adegenet* in R environment [84] (**Fig 1B** in main text). To avoid over-fitting the model, we used the cross validation method to choose the optimum number of principal components to include in the model. We retained 15 principal components and 4 discriminant factors in the final model, which explained 57% of the sample variability. To find the number of genetic groups better fitting our sample, we used the k-means algorithm for a number of groups k=1–25. We ran 10^9 iterations with 2,000 starting points. The Bayesian Information Criterion (BIC) minimized at k=12 for the whole sample analysis (**Figs C** and **D**).

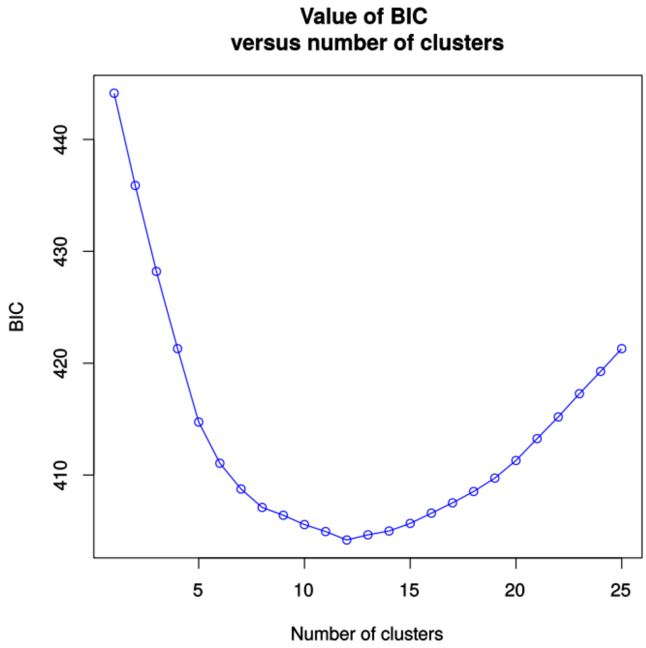

Fig C. Inference of number of clusters in DAPC. The Bayesian Information Criterion (BIC) is minimum at k=12, suggesting an optimal separation of samples in 12 groups.

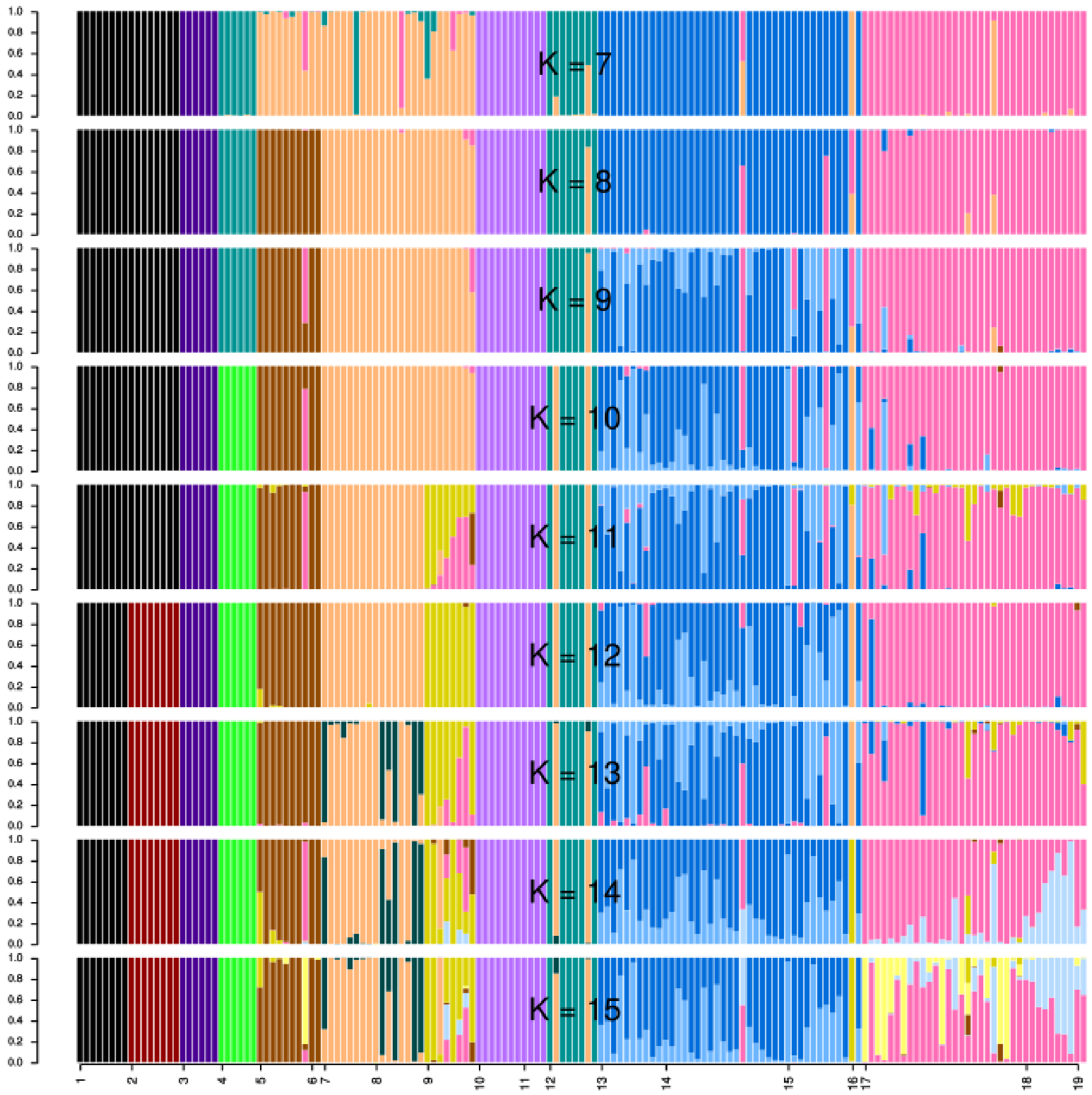

Fig D. Individual assignment probability to each group from Discriminant Analysis of Principal Components (DAPC). Horizontal axis shows the population codes as in **Table B**. Vertical axis shows the assignment probabilities for values of k from 7 to 15. Colors for k=12 are the same as in **Fig 1B** in main text.

We also used a Bayesian clustering approach implemented in the program STRUCTURE v2.3.4 using the correlated-frequencies model without admixture [67] (**Fig E**). We assessed the structure of the sample for a number of clusters k=7–15. For each value of k, we ran 10 repetitions of 2×10^6 iterations of the MCMC algorithm after discarding 5×10^5 iterations as burn-in. Finally, we performed a Principal Component Analysis with the *adegenet* package [64) (**Fig F**).

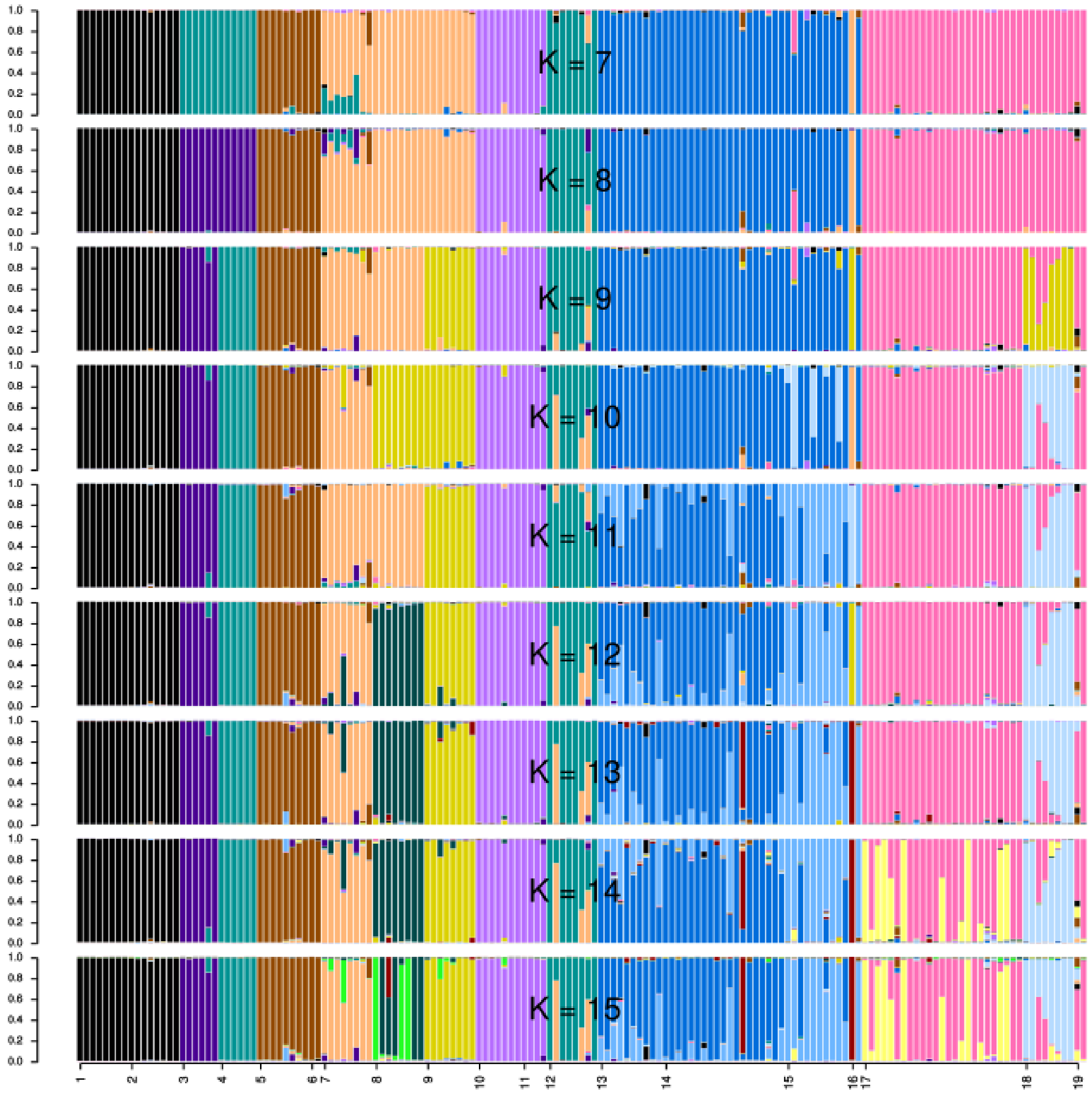

Fig E. Individual assignment probability to each group from STRUCTURE. Horizontal axis shows the population codes as in **Table B**. Vertical axis shows the assignment probabilities for values of k from 7 to 15. For each value of k only the run with highest likelihood is represented. Colors for k=12 are the same as in **Fig F** showing the results from PCA.

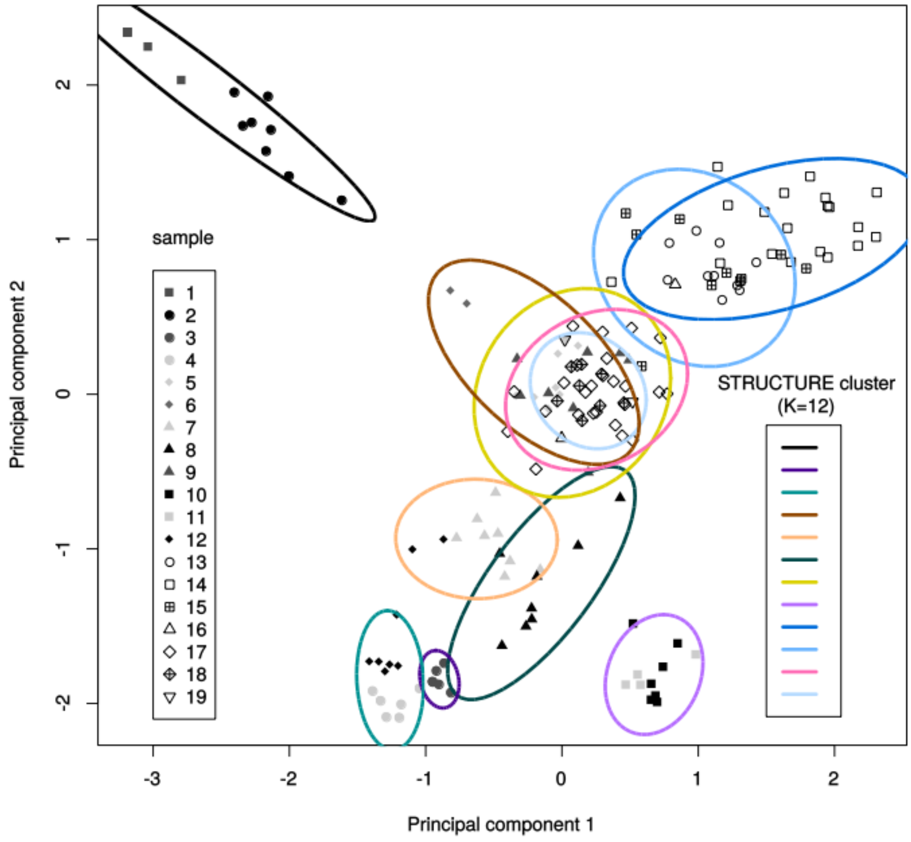

Fig F. First two principal components for the Principal Component Analysis (PCA). Colored ellipses show the genetic groups identified with STRUCTURE for k=12 (see **Fig E**).

Coalescent-based analyses. An approximate Bayesian computation (ABC) approach using random forests [68,69] was used to characterize the local demographic history of quinoa found around Antofagasta de la Sierra. The coalescence-based modeling of genetic relationships between modern and ancient samples from Antofagasta examined six scenarios (**Fig 2** in main text): i) direct chronological filiation between all samples, mixing cultivated and wild forms (**Fig 2A**), ii) replacement of all ancient quinoas by modern quinoas (**Fig 2B**), iii) filiation between modern quinoas and intermediate ancient quinoas, both replacing the oldest quinoas (**Fig 2C**), iv) successive replacement of the three groups of quinoas: modern, intermediate, ancient (**Fig 2D**), v) same scenario as previously but differentiating between cultivated and wild forms (**Fig 2E**), vi) same scenario as previously but with admixture between cultivated and wild forms (**Fig 2F**).

Coalescent simulations and calculation of summary statistics were performed with DIYABC [85]. Reference tables were exported and ABC model choice and parameter estimation was performed with the random forest approach implemented in the R package *abcrf* [86]. The scripts in R employed are available at the Zenodo open access repository (https://zenodo.org/*)*. All single-sample and two-sample summary statistics available at DIYABC and admixture summary statistics (from a reduced number of relevant sample trios) were used to grow random forests for ABC. **Table G** presents prior and posterior probability distributions for parameters of the models. For each scenario 60 000 simulations were performed. Random forests of 800 trees were grown for model choice. For the best model, 140 000 additional simulation were run and random forests of 1000 trees were grown for parameter estimation.

Table G. Prior and posterior probability distribution of model parameters for the approximate Bayesian computation analysis.

Parameter	Prior distribution	conditions	Posterior (median and 95%HPD)*
Age sample #12**	Constant=-56		
Age sample #13 (t_1)**	Normal (m=690, σ=50)		Indistinguishable from prior
Age samples #14–15 (t_2)**	Normal (m=796, σ=24)	$t_2>t_1$	Indistinguishable from prior
Age samples #17–#18 (t_3)**	Normal (m=1364, σ=20)	$t_3>t_2$	Indistinguishable from prior
Age sample #19 (t_4)**	Normal (m=1796, σ=23)	$t_4>t_3$	Indistinguishable from prior
Admixture proportion from wild genetic pool for sample #15 (α_1)	Uniform (min=0, max=1)		0.35 (0.18–0.69)
Admixture proportion from wild genetic pool for sample #18 (α_2)			0.48 (0.30–0.82)
Time of divergence (td_1), between clusters	Uniform (min=2000, max=7000)	$td_2>td_1$	Indistinguishable from prior
Time of domestication (td_2), domesticated and wild	Uniform (min=5000, max=7000)		Indistinguishable from prior
Effective population size (Na_0), 'Green' cluster (sample #12)	Log-Uniform (min=2, max=10^6)		185 (37–1231)
Effective population size (Nb_0), 'Blue' cluster (samples #13–15) between t_1 and t_2			866 (133–64026)
Effective population size (Nb_1), 'Blue' cluster (samples #13–15) between t_2 and td_1			857 (195–2981)
Effective population size (Nc_0), 'Red' cluster (samples #17–19) between t_3 and t_4			1469 (535–7587)
Effective population size (Nc_1), 'Red' cluster (samples #17–19) between t_4 and td_1			1035 (130–116845)
Effective population size (N_W), wild population			30 (2–3094)
Other effective population size parameters (e.g. ancestral populations)			Indistinguishable from prior
Mutation rate (μ)	Log-Uniform (min=10^{-5}, max=10^{-2})		1.10×10^{-3} (8.18×10^{-5}–5.59×10^{-3})
Geometric distribution parameter for GSM (P_{GSM})	Uniform (min=0, max=1)		0.60 (0.09–0.98)
Mutation rate SNI (μ_{SNI})	Log-Uniform (min=10^{-9}, max=10^{-5})		Indistinguishable from prior

* Only reported posterior probability estimates that differ conspicuously from prior probability distributions.

** Time measured in years before present, 'present' being set to calendar year 1950 to follow scale of radiocarbon dating.

References for Supporting Information

References 1 to 69 are in Reference list in the main text.

70. Hocsman S. Producción lítica, variabilidad y cambio en Antofagasta de la Sierra (ca. 5500-2000 AP) [Doctoral Thesis]. La Plata, Argentina: FCNyM-Universidad Nacional de La Plata; 2006.

71. Paabo S, Poinar H, Serre D, Jaenicke-Despres V, Hebler J, Rohland N, et al. Genetic analyses from ancient DNA. Annu Rev Genet. 2004;38:645-79.

72. O'Donoghue K, Clapham A, Evershed RP, Brown TA. Remarkable preservation of biomolecules in ancient radish seeds. Proc Roy Soc Lond B. 1996;263:541-7.

73. Lia VV, Confalonieri VA, Ratto N, Hernandez JAC, Alzogaray AMM, Poggio L, et al. Microsatellite typing of ancient maize: insights into the history of agriculture in southern South America. Proc Roy Soc Lond B. 2007 Feb;274:545-54. PubMed PMID: ISI:000243354200012.

74. Wilson AS, Taylor T, Ceruti MC, Chavez JA, Reinhard J, Grimes V, et al. Stable isotope and DNA evidence for ritual sequences in Inca child sacrifice. Proc Natl Acad Sci USA. 2007;104:16456-61.

75. Burrieza HP, Sanguinetti A, Michieli CT, Bertero HD, Maldonado S. Death of embryos from 2300-year-old quinoa seeds found in an archaeological site. Plant Sci. 2016;253:107-17.

76. Mason SL, Stevens MR, Jellen EN, Bonifacio A, Fairbanks DJ, Coleman CE, et al. Development and use of microsatellite markers for germplasm characterization in quinoa (*Chenopodium quinoa* Willd.). Crop Science. 2005;45:1618-30.

77. Jarvis D, Kopp O, Jellen E, Mallory M, Pattee J, Bonifacio A, et al. Simple sequence repeat marker development and genetic mapping in quinoa (*Chenopodium quinoa* Willd.). Journal of Genetics. 2008;87:39-51.

78. Thompson JD, Higgins DG, Gibson TJ. CLUSTAL W: improving the sensitivity of progressive multiple sequence alignment through sequence weighting, position-specific gap penalties and weight matrix choice. Nucleic Acids Res. 1994;22:4673-80.

79. Meyers BC, Tingley SV, Morgante M. Abundance, distribution, and transcriptional activity of repetitive elements in the maize genome. Genome Res. 2001;11:1660-76.

80. Hurlbert SH. Nonconcept of species diversity - critique and alternative parameters. Ecology. 1971;52:577-86.

81. Weir BS, Cockerham CC. Estimating F-statistics for the analysis of population-structure. Evolution. 1984;38:1358-70.

82. Hartl DL, Clark AG. Principles of Population Genetics. Fourth Edition. Sunderland, MA, USA: Sinauer Associates; 2007.

83. David P, Pujol B, Viard F, Castella V, Goudet J. Reliable selfing rate estimates from imperfect population genetic data. Mol Ecol. 2007;16:2474-87.

84. Jombart T, Devillard S, Balloux F. Discriminant analysis of principal components: a new method for the analysis of genetically structured populations. Bmc Genetics. 2010;11:94.

85. Cornuet J-M, Pudlo P, Veyssier J, Dehne-Garcia A, Gautier M, Leblois R, et al. DIYABC v2.0: a software to make approximate Bayesian computation inferences about population history using single nucleotide polymorphism, DNA sequence and microsatellite data. Bioinformatics. 2014;30:1187-9.

86. Marin JM, Raynal L, Pudlo P, Robert CP, Estoup A. abcrf: Approximate Bayesian computation via random forests. R package version 17. 2017.
