# Supplementary material for: Discontinuities in quinoa biodiversity in the dry Andes: An 18-century perspective based on allelic genotyping
Source: PLoS One. 2018 Dec 5;13(12):e0207519. doi: 10.1371/journal.pone.0207519 (PMC6281180; doi:10.1371/journal.pone.0207519)
Supplement: S1 File — S1–File-WINKEL-et-al-Allelic-profiles-ReadMe. (PDF) [file pone.0207519.s002.pdf]

WINKEL T. et al. Discontinuities in quinoa biodiversity in the dry Andes: an 18-century perspective based on allelic genotyping

S2. SEED SAMPLE ALLELIC PROFILES

Lines 1-2: General dataset descriptors
Number of loci, sample seeds, populations, individual seeds per population

Line 3: Numeric codes and alphanumeric names of the 19 populations

Line 4: Column headings
Sample seed name, sample seed type (modern/archaeological), locus

Lines 5-161: Sample seed allelic profile
base pair numbers; missing data code: -1
